# Effect of Diterpenes Isolated of the Marine Alga *Canistrocarpus cervicornis* against Some Toxic Effects of the Venom of the *Bothrops jararaca* Snake

**DOI:** 10.3390/molecules20033515

**Published:** 2015-02-18

**Authors:** Thaisa Francielle Souza Domingos, Magui Aparecida Vallim, Diana Negrão Cavalcanti, Eládio Flores Sanchez, Valéria Laneuville Teixeira, André Lopes Fuly

**Affiliations:** 1 Department of Molecular and Cellular Biology, Institute of Biology, Federal Fluminense University, Niteroi 24020-141, RJ, Brazil; E-Mail: thaisadomingos@yahoo.com.br; 2 Department of Marine Biology, Institute of Biology, Federal Fluminense University, Niteroi 24020-141, RJ, Brazil; E-Mails: mvallim@yahoo.com.br (M.A.V.); dn.cavalcanti@gmail.com (D.N.C.); valerialaneuville@gmail.com (V.L.T.); 3 Ezequiel Dias Foundation, Belo Horizonte 30510-010, MG, Brazil; E-Mail: eladiooswaldo@gmail.com

**Keywords:** *Canistrocarpus cervicornis*, marine alga, *Bothrops jararaca*, snake venom, toxic activities, inhibition, bioprospecting

## Abstract

Snake venoms are composed of a complex mixture of active proteins and peptides which induce a wide range of toxic effects. Envenomation by *Bothrops jararaca* venom results in hemorrhage, edema, pain, tissue necrosis and hemolysis. In this work, the effect of a mixture of two secodolastane diterpenes (linearol/isolinearol), previously isolated from the Brazilian marine brown alga, *Canistrocarpus cervicornis*, was evaluated against some of the toxic effects induced by *B. jararaca* venom. The mixture of diterpenes was dissolved in dimethylsulfoxide and incubated with venom for 30 min at room temperature, and then several *in vivo* (hemorrhage, edema and lethality) and *in vitro* (hemolysis, plasma clotting and proteolysis) assays were performed. The diterpenes inhibited hemolysis, proteolysis and hemorrhage, but failed to inhibit clotting and edema induced by *B. jararaca* venom. Moreover, diterpenes partially protected mice from lethality caused by *B. jararaca* venom. The search for natural inhibitors of *B. jararaca* venom in *C. cervicornis* algae is a relevant subject, since seaweeds are a rich and powerful source of active molecules which are as yet but poorly explored. Our results suggest that these diterpenes have the potential to be used against Bothropic envenomation accidents or to improve traditional treatments for snake bites.

## 1. Introduction

Envenomation caused by snake bites is a public health problem, mainly in tropical and poor countries, and because of the high incidence of deaths and sequelae derived from such accidents, the World Health Organization (WHO) considers it a neglected health issue [1,2]. Snake venoms are a complex mixture of proteins, including phospholipases A_2_ (PLA_2_s), proteases, myotoxins, nucleotidases and others, that are directly involved in local damage (myonecrosis, hemorrhage and edema) as well as in systemic effects (bleeding, coagulopathy, renal failure and shock) [3,4]. In Brazil, most accidents (90%) are caused by the genus Bothrops, and *B. jararaca* causes the highest number of incidents (95%) [5,6]. The regular treatment available for these accidents is the administration of a monovalent or polyvalent antibothropic serum. Although serotherapy effectively counteracts the systemic actions of venoms, such treatment has some disadvantages including poor availability for distant regions, short expiration date, strong potential for allergic reactions [7,8] and low effectiveness against the local effects of venoms [9]. Moreover, delay in administering the serum may further reduce its effectiveness, leading to irreversible and disabling damage [10]. Due to the limitation of the treatment, searches for alternative venom inhibitors, either synthetic or natural, to complement the action of currently utilized antivenoms, is of great interest.

The oceans are a source of many living organisms that produce a variety of metabolically derived molecules classified as primary and secondary metabolites. A group of metabolites that deserves special attention are the terpenes. Besides their ecological functions (defense against predators, competitors and herbivores) [11], they display pharmacological effects, such as antimicrobial [12], antiviral [13], anticancer [14], inhibition of caterpillar [15] and snake [16] venoms as well as anticoagulation [17], antimalarial and antituberculous effects [18]. A variety of terpenes have been isolated from Dictyotacea species marine brown algae [19], and some pharmacological and ecological activities of dolastane and secodolastane diterpenes isolated from *C. cervicornis* (formerly *Dictyota cervicornis*) have been investigated [16,20]. In our previous work, we showed that dolastane and two secodolastane diterpenes (linearol/isolinearol) isolated from the marine brown alga, *C. cervicornis*, displayed antivenom effects against some toxic activities of *Lacheis muta* [16,21]. In the present work, we evaluated the effect of these two secodolastane diterpenes isolated from the same alga to inhibit some *in vitro* (hemolysis, proteolysis and clotting) and *in vivo* (hemorrhage, edema and lethal) activities of *B. jararaca* venom.

## 2. Results and Discussion

Marine organisms are among the richest sources of natural products, with a variety of properties that have led to their use by human beings as sources of medicines and food, as well as for biotechnological applications [22,23,24]. In Brazil, the brown marine algae are the most widely studied, with more than 300 diterpenes isolated from at least 35 species all over the world [19]. Although some biological activities have been studied [21,25,26,27], the potential of brown seaweeds to neutralize the toxic effects of snake venoms has not been widely investigated.

### 2.1. Antihemolytic and Antiproteolytic Effect

**Figure 1:**
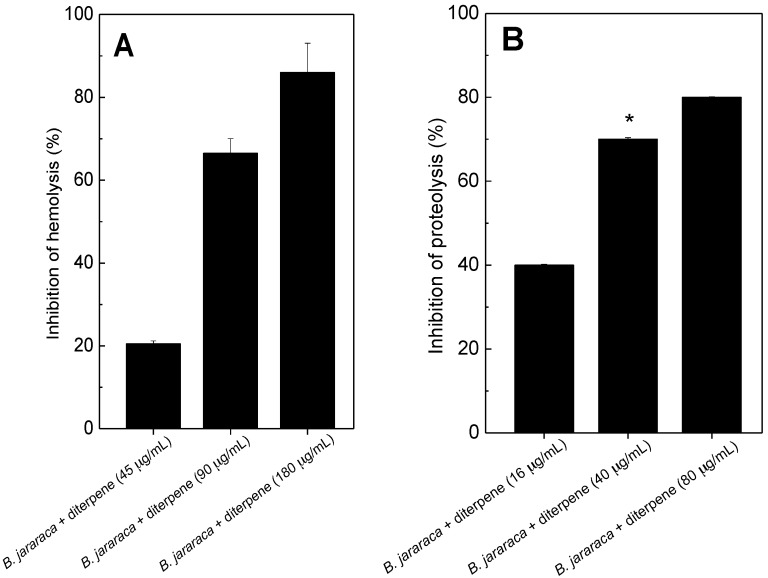
(**A**) *B. jararaca* venom (18 µg/mL) was incubated for 30 min at room temperature with a mixture of diterpenes at 45 µg/mL, 90 µg/mL or 180 µg/mL; and (**B**) *B. jararaca* venom (2 µg/mL) was incubated with diterpenes at 16 µg/mL, 40 µg/mL or 80 µg/mL, and then hemolytic (A) and proteolytic (B) activities were measured, as described in the Experimental Section. Data are expressed as mean ± SEM of three individual experiments (n = 3). * *p* < 0.05 when compared to first column.

The mixture of diterpenes linearol/isolinearol inhibited hemolysis (Figure 1A) and proteolysis (Figure 1B) caused by *B. jararaca* venom in a concentration-dependent manner. Neither the single diterpenes nor dimethylsulfoxide (DMSO, the vehicle) induced or inhibited hemolysis or proteolysis. PLA_2_ enzymes are one of the most active components in snake venoms [28]. These enzymes induce a wide range of pharmacological and toxic effects, including hemolysis, neurotoxicity, cardiotoxicity, effects on platelet aggregation, myotoxicity and edema, that contribute to the envenomation symptoms [29,30]. Domingos *et al.* [19] have reported the ability of the mixture of diterpenes (linearol/isolinearol) to inhibit the crude venom as well as a PLA_2_ isolated from *L. muta* venom; but, its mechanism of action has not been investigated yet. Moreover, other reports have cited PLA_2_ inhibitors from marine organisms, and some of them are terpenes [31].

### 2.2. Anticoagulation

**Figure 2:**
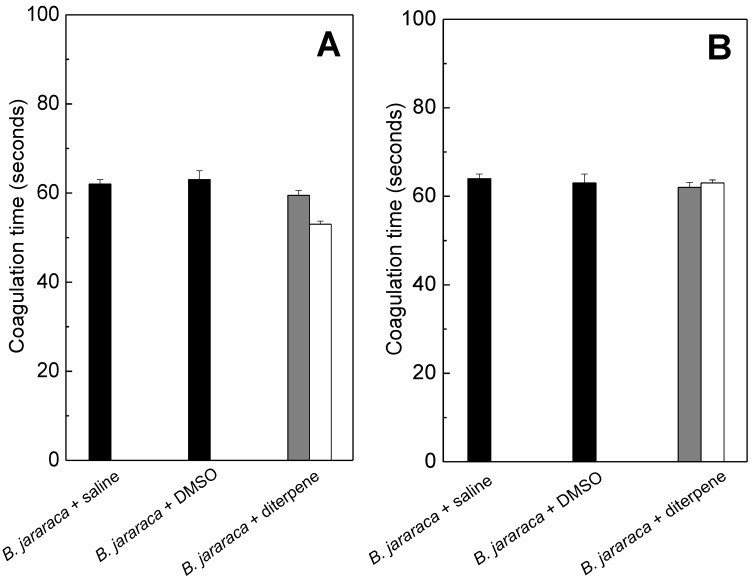
*B. jararaca* venom (12 µg/mL) was incubated for 30 min at room temperature with saline (1), DMSO (1% v/v) (2) or diterpenes at 360 µg/mL (gray columns) (3) or at 720 µg/mL (white columns, 3). Then, the mixture was added to plasma (**A**) or to fibrinogen (**B**), and coagulation time was recorded. Data are expressed as mean ± SEM of three individual experiments (n = 3).

*B. jararaca* venom induces plasma clotting that it is associated with the action of a specific group of enzymes, the metallo- and serine enzymes. The snake venom serine proteinases (SVSPs) are among the best-characterized enzymes to affect the hemostatic system [32,33,34]. Some may affect platelet function or disrupt specific factors of the coagulation cascade, causing an imbalance in the hemostatic system of the victim [35]. As seen in Figure 2, the mixture of linearol/isolinearol (360 and 720 µg/mL) did not inhibit plasma clotting or fibrinogen triggered by *B. jararaca* venom, regardless of the concentration of diterpenes tested.

### 2.3. Antihemorrhagic and Antiedematogenic Activities

**Figure 3:**
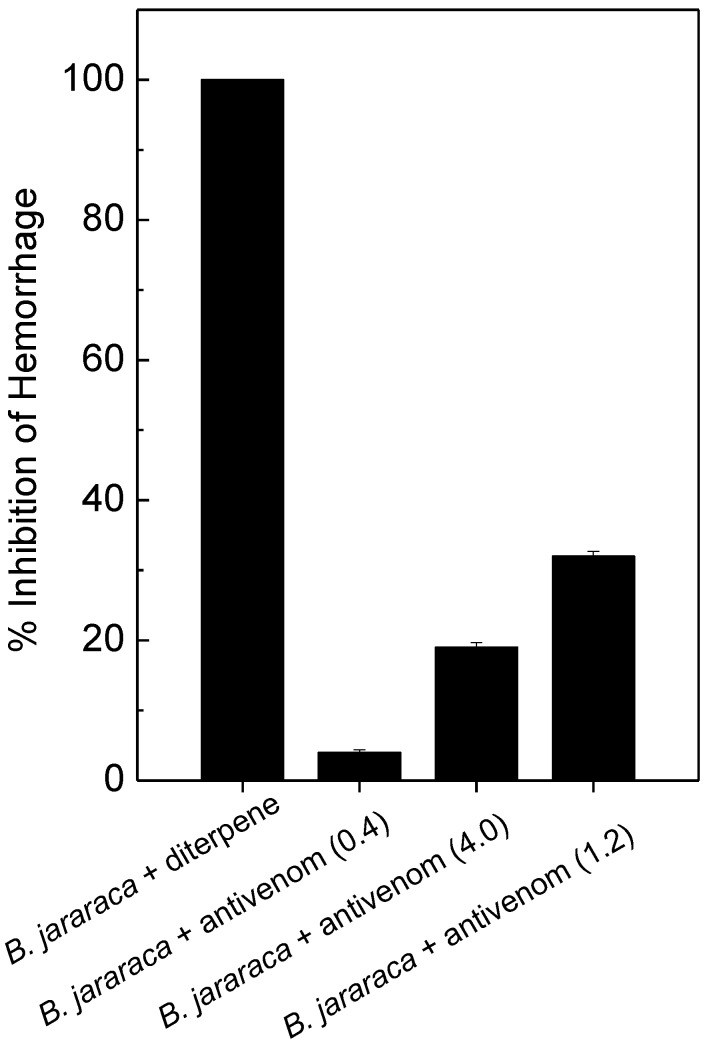
*B. jararaca* venom (3 µg/g) was incubated for 30 min at room temperature with a mixture of diterpenes (87 µg/mL) or with antibothropic serum (antivenom) at 0.4 mL/mg, 1.2 mL/mg or 4.0 mL/mg. Then, hemorrhagic activity was assayed, as described in the methods. Data are expressed as mean ± SEM of two individual experiments (n = 4).

The mixture of linearol/isolinearol (87 µg/g) inhibited 100% of the hemorrhagic activity of *B. jararaca* venom (at 3 µg/g, Figure 3). Snake venom metalloproteinases (SVMPs) are zinc-dependent enzymes that cleave, in a highly selective fashion, key peptide bonds of some plasma and extracellular matrix proteins, leading to hemorrhage [35]. Besides hemorrhage, SVMPs activate blood clotting, have apoptotic activity, inhibit platelet aggregation and are pro-inflammatory [34]. According to proteomic approaches, SVMPs represent the major components of snake venoms [34] and contribute to the toxicity of venoms. The inhibition of hemorrhagic activity may suggest an interaction between diterpenes with Zn^2+^ which is located at the active site of such enzymes [36], thus blocking the hemorrhagic effect. 

Moreover, another experimental protocol was employed in order to mimic a real envenomation situation, whereby. *B. jararaca* venom (3 µg/g) was injected subcutaneously (s.c.), and 20 min later the diterpenes (87 µg/g) were injected at the same site where the venom was injected. Again, *B. jararaca*-induced hemorrhage was inhibited by around 80% (data not shown). This result is very interesting, since hemorrhage is one of the main effects responsible for amputations or deformity in envenomed victims. On the other hand, at the highest antibothropic serum dose (4 mL/mg), hemorrhagic activity of *B. jararaca* was only partially inhibited (30%, Figure 3). This low antivenom efficacy to neutralize hemorrhage caused by Bothrops venom has been described elsewhere [9,30].

Envenomation by snakes usually promotes an intense inflammatory reaction and edema, and PLA_2_ enzymes and other enzymes are responsible for such effects. The diterpenes (87 µg/g) did not inhibit edematogenic activity of *B. jararaca* venom (at 2 µg/g, data not shown); and thus, would not prevent inflammation induced by snake bites. On the other hand, in our previous work, this mixture of diterpenes inhibited hemolysis and edema induced by a PLA_2_ isolated from *L. muta* [21]. Despite the similarity in composition between the *B. jararaca* and *L. muta* venoms, the presence of specific components in each venom may explain the different inhibitory profiles of the diterpenes.

### 2.4. Antilethality Activity 

Injection of *B. jararaca* venom (30 µg/g) incubated either with saline or DMSO killed all mice after around 330 min (Table 1). When diterpenes (60 µg/g) were incubated with *B. jararaca* venom, all animals died around the same time period as well. However, the animals that received antibothropic serum, at any concentration (0.4, 1.2 or 4 mL/mg), did not die (Table 1). Therefore, the diterpenes did not protect mice from the toxic effects of *B. jararaca* venom, unlike the antibothropic serum used. Such results do not discourage the use of diterpenes as antivenom agents, since the mixture of diterpenes, unlike the antiserum, fully inhibited local effects (hemorrhage and proteolysis); that are responsible for amputation and morbidity.

**Table 1:** Effect of mixture of diterpenes on lethality of *B. jararaca* venom. *B. jararaca* venom (30 µg/g) was incubated for 30 min at room temperature with 60 µg/g of diterpenes or with 0.4 mL/mg, 1.2 mL/mg or 4.0 mL/mg of antibothropic serum. Control experiments were performed by incubating *B. jararaca* venom with saline or DMSO. Also, diterpenes were mixed with saline. Then, mixtures were injected i.p. into mice, and death of animals was observed. ND, means Not Died.

Groups	Death (min)
*B. jararaca* + saline	320 ± 14
*B. jararaca* + DMSO	350 ± 21
Saline + diterpenes	ND
*B. jararaca* + diterpenes	310 ± 20
*B. jararaca* + antibothropic serum (0.4 mL/mg)	ND
*B. jararaca* + antibothropic serum (1.2 mL/mg)	ND
*B. jararaca* + antibothropic serum (4.0 mL/mg)	ND

## 3. Experimental Section

### 3.1. Algal Material

Specimens of *C. cervicornis* (Dictyotaceae, *Phaeophyta*) were collected during May, 2006, at Praia do Forno, in the city of Armação de Búzios, located in the north of Rio de Janeiro State (22°45′42″S and 41°52′27″W), Brazil, at depths ranging from 0.3 to 2 m. The seaweeds were washed with local sea water, separated from sediments, epiphytes and other associated organisms. Voucher specimens were deposited at the herbarium of the Universidade do Estado do Rio de Janeiro (HRJ 10754).

### 3.2. Isolation of Diterpenes

The air-dried algal material (100 g) was extracted in dichloromethane (CH_2_Cl_2_, 100%) at room temperature for seven days, yielding 14 g of a crude extract. The diterpenes 4-hydroxy-9,14-dihydroxydolasta-1(15),7-diene (isolinearol, **1**) and the 2-hydroxy-9,14-dihydroxydolasta-1(15),7-diene (linearol, **2**) were isolated from the CH_2_Cl_2_ crude extract of *C. cervicornis* after chromatography on a silica gel column. The fractions eluted with 100% CH_2_Cl_2_ contained the dolastane diterpene **1**. The fractions eluted with 4:1 CH_2_Cl_2_/EtOAc yielded a mixture of the two secodolastane diterpenes **1** and **2** (35 mg), respectively, in a proportion of 3:2. The compounds **1** and **2** were identified by comparison of their physical and spectroscopic data with reported values [13,27,37]. Finally, the diterpenes were dissolved in DMSO to perform the biological assays.

### 3.3. Snake Venom, Antivenom and Animals

Lyophilized *B. jararaca* venom and monovalent antibothropic serum were provided by the Ezequiel Dias Foundation (FUNED), Belo Horizonte, MG, Brazil. Balb/c mice (weighing 18–20 g) were obtained from the Núcleo de Animais de Laboratório of the Universidade Federal Fluminense (protocol number 200/10). They were housed under controlled temperature (24 ± 1 °C) and light conditions, and all of the performed experiments were approved by the UFF Institutional Committee for Ethics in Animal Experimentation and were in accordance with the guidelines of the Brazilian Committee for Animal Experimentation (COBEA).

### 3.4. Antihemolytic Activity

The degree of hemolysis of *B. jararaca* venom was determined by the indirect hemolytic test using human erythrocytes and hen’s egg yolk emulsion as substrate [38]. The amount of *B. jararaca* venom (μg/mL) that produced 100% hemolysis was designed as the Minimum Indirect Hemolytic Dose (MIHD). Inhibitory experiments were determined by incubating the mixture of diterpenes (linearol/isolinearol) with one MIHD for 30 min at room temperature, and then hemolytic activity was assayed.

### 3.5. Anticoagulant Activity

The clotting of human plasma or fibrinogen induced by *B. jararaca* venom was determined on a digital Amelung Model KC4A coagulometer (Labcon, Bavaria, Germany). Different concentrations of *B. jararaca* venom were mixed with fibrinogen solution (2 mg/mL, final concentration) or with human plasma (donated from healthy volunteers of the local blood bank of the Hospital of the Federal Fluminense University), and the amount of venom (μg/mL) that clotted either fibrinogen or plasma in 60 s was designed as the Minimum Coagulant Dose (MCD). To evaluate the inhibitory effect, the diterpenes were incubated for 30 min at room temperature with one MCD of venom, and then, the mixture was added to fibrinogen or plasma and clotting time recorded. Control experiments were performed in parallel by mixing DMSO (0.5% v/v, final concentration) or saline with venom, instead of diterpenes.

### 3.6. Antiproteolytic Activity

Proteolytic activity of *B. jararaca* venom was determined using azocasein as substrate (0.2% w/v, in 20 mM Tris-HCl, 8 mM CaCl_2_, pH 8.8), with minor modification [39]. An Effective Concentration (EC) was defined as the amount of venom (μg/mL) able to produce a variation of about 0.2 OD units at *A* 420 nm. The diterpenes were incubated with one EC of *B. jararaca* venom for 30 min at room temperature and then proteolytic activity was measured.

### 3.7. Antihemorrhagic Activity

Hemorrhagic lesions produced by *B. jararaca* venom were quantified using a procedure described elsewhere [40], with minor modifications. Briefly, samples were injected subcutaneously (s.c) into the abdominal skin of mice. Two hours later, the animals were euthanized, the abdominal skin removed, stretched and inspected for visual changes in the inner layer in order to localize hemorrhagic spots. Hemorrhage was expressed as Minimum Hemorrhagic Dose (MHD), defined as the amount of venom (µg/g) able to produce a hemorrhagic halo of 10 mm [41]. The effect of diterpenes was investigated by incubating them with one MHD of *B. jararaca* venom for 30 min at room temperature. Then, the mixture of diterpenes was injected s.c. into mice and hemorrhage measured. Hemorrhagic activity was expressed as the mean diameter (in millimeters) of the hemorrhage halo induced by *B. jararaca* venom in the absence and presence of the diterpenes. Antibothropic serum 0.4, 1.2, 4.0 mL/mg was also incubated with venom, and the mixture was injected s.c. into mice. In another set of experiment, *B. jararaca* venom was injected s.c., and 20 min later, the diterpenes were injected at the same site of venom injection. Negative controls were performed by injecting s.c. DMSO (0.9% v/v, final concentration) or saline. The total number of mice for each experimental group was 4–5, and experiments were repeated twice.

### 3.8. Antiedematogenic Activity

The edema-inducing activity of *B. jararaca* venom was determined according to Yamakawa *et al.* [42], with modifications. Groups of mice received 50 µL of venom subcutaneously (s.c) in the right foot pad, whereas the left food pad received 50 µL of saline or DMSO. One hour after injection, edema was evaluated and expressed as the percentage of increase in the weight of the right foot pad compared to the left one. The inhibitory effect was investigated by incubating diterpenes with *B. jararaca* venom for 30 min at room temperature, and then the mixtures were injected s.c. into mice and edema was measured. Negative controls were performed by mixing venom with DMSO (0.9% v/v, final concentration) or with saline. The total number of mice for each experimental group was 4–5, and experiments were repeated twice.

### 3.9. Antilethal Activity

Different doses *B. jararaca* venom were injected intraperitoneally (i.p) into groups of mice and the number of deaths for each dose of venom was observed over a period of 24 to 48 h. For neutralization assays, the diterpenes were incubated for 30 min at room temperature with *B. jararaca* venom, and then, the mixture was injected i.p. and lethal activity was assessed. The lethality was determined and compared with the control group that received *B. jararaca* venom. Antibothropic serum 0.4, 1.2, 4.0 mL/mg was also incubated with venom, and the mixture was injected i.p. into mice. In control experiments, DMSO (0.9% v/v, final concentration) or saline was incubated with venom instead of diterpenes, and injected i.p. into mice. The total number of mice for each experimental group was 5–6, and experiments were repeated twice.

### 3.10. Statistical Analysis

Results are expressed as mean ± SEM obtained with the indicated number of animals or experiments performed. The statistical significance of differences among experimental groups was evaluated using the Student’s t test and p values of ≤0.05 were considered statistically significant.

## 4. Conclusions

Our results suggest that diterpenes from the marine alga, *C. cervicornis*, are a promising source of molecules to improve the regular treatment for envenomation by *B. jararaca* snake bites, and/or useful as prototypes for designing alternative/new antiophidian molecules. However, an in-depth scientific investigation is imperative to evaluate the antivenom potential of natural products in order to derive therapeutically effective natural products for snake bites.
